# *Zingiber officinale* Leaf Subcritical Water Extract Suppresses Adipogenesis and Lipogenesis in 3T3-L1 Adipocytes via the AMPK-SREBP-1c Signaling Pathway

**DOI:** 10.7150/ijms.121198

**Published:** 2026-06-04

**Authors:** Eun-Min Jun, Jong-Yeon Kim, Jeong-Sook Choe, Eun-Jung Park, Hae-Jeung Lee

**Affiliations:** 1 Department of Food and Nutrition, Gachon University, Seongnam 13120, Republic of Korea.; 2 Institute for Aging and Clinical Nutrition Research, Gachon University, Seongnam 13120, Republic of Korea.; 3 Rural Development Administration, Jeonju 54875, Republic of Korea.; 4 Department of Health Sciences and Technology, GAIHST, Gachon University, Incheon 21999, Republic of Korea.; 5 Gachon Biomedical Convergence Institute, Gachon University Gil Medical Center, Incheon 21565, Republic of Korea.

**Keywords:** Ginger leaf, Subcritical water, 3T3-L1, Adipogenesis, Lipogenesis, AMPK-SREBP-1c

## Abstract

*Zingiber officinale*, commonly known as ginger, has long been utilized as a medicinal plant due to its diverse pharmacological properties. However, research has predominantly focused on the rhizome, while the leaf is frequently regarded as a byproduct. In this study, we investigated the functional relevance of the ginger leaf, concentrating on the anti-obesity effects of ginger leaf subcritical water extract (GLE) and the underlying mechanisms in 3T3-L1 adipocytes. GLE reduced lipid accumulation in a concentration-dependent manner on day 7 of differentiation and suppressed the expression of adipogenesis-associated factors, including CCAAT/enhancer-binding protein α (C/EBPα), peroxisome proliferator-activated receptor γ, fatty acid binding protein 4, and lipoprotein lipase on both days 2 and 7. GLE also modulated early adipogenesis by inhibiting p38 phosphorylation and downregulating C/EBPβ expression at 4 h of differentiation. Additionally, GLE decreased the expression of acetyl-CoA carboxylase, fatty acid synthase, and stearoyl-CoA desaturase 1—key enzymes involved in lipogenesis—from the early stages of adipocyte differentiation. Furthermore, GLE directly phosphorylated adenosine monophosphate-activated protein kinase (AMPK), leading to inhibition of the expression of sterol regulatory element-binding protein-1c (SREBP-1c), an upstream regulator of both adipogenesis and lipogenesis. In summary, GLE exerted anti-obesity effects in 3T3-L1 adipocytes by inhibiting the transcriptional activation of adipogenic and lipogenic regulators during the early stage of differentiation, and maintaining their downregulation at later stages, and it was found to be mediated by the AMPK-SREBP-1c signaling pathway.

## Introduction

Obesity is commonly described as excessive body fat accumulation, and weight gain occurs when energy intake chronically exceeds energy expenditure 1. Dysfunctional and hypertrophic adipose tissue can secrete adipose-derived factors and contribute to obesity-associated complications, including type 2 diabetes, cancer, and cardiovascular diseases 2, 3. The prevalence of obesity has risen markedly worldwide 4, 5, particularly in Korea, where obesity prevalence has increased 1.27-fold since 2012 6. Consequently, interest in pharmacotherapy for obesity treatment is increasing 7. However, many anti-obesity drugs have been withdrawn due to severe adverse effects 8. Consequently, the demand for natural products with lower toxicity, such as herbal remedies, is expanding.

*Zingiber officinale*, commonly known as ginger, is a perennial herb of the Zingiberaceae family and contains approximately 80-90 non-volatile bioactive compounds, including gingerol, shogaol, quercetin, ferulic acid, rutin, and catechin 9-11. These compounds exhibit a broad spectrum of pharmacological activities, such as anti-obesity, antioxidant, and anti-inflammatory effects, and ginger has been used medicinally since ancient times 12. For example, boiled ginger improved the lipid profile in rats fed a high-cholesterol diet 13, and another study indicated that ginger may prevent obesity via multiple mechanisms, including thermogenesis, lipolysis, lipogenesis, fat absorption, and controlling appetite 14. Notably, recent research demonstrated that among ginger plant parts, the leaf contains the highest levels of polyphenols such as rutin, quercetin-3-glucoside, quercetin, and kaempferol 15. Despite this, most studies have concentrated on the rhizome, while the leaf is typically discarded during cultivation and remains underutilized despite its biofunctional potential 16. Therefore, investigating the biofunctional properties of ginger leaves and identifying novel applications is warranted.

The growth and maturation of preadipocytes proceed through sequential processes, including adipogenesis, lipogenesis, and lipid accumulation. In particular, adipogenesis proceeds through several stages. Following hormonal stimulation, preadipocytes undergo growth arrest and several rounds of cell division, which is mitotic clonal expansion (MCE; 0-2 days) 17. Upon completion of MCE, cells enter terminal differentiation (2-7 days), during which late adipogenic markers, along with lipogenic genes, are upregulated 18-21. Adenosine monophosphate-activated protein kinase (AMPK) and sterol regulatory element-binding protein 1c (SREBP-1c) act as upstream regulators of these processes. SREBPs serve as master regulators of lipid biosynthesis by controlling enzymes involved in the synthesis of cholesterol, fatty acids (FAs), and triglycerides (TGs) 22, 23. In mammals, SREBPs consist primarily of isoforms SREBP-1a, -1c, and -2, with SREBP-1c predominantly expressed in liver and adipose tissue 24. AMPK is present in all tissues and maintains energy homeostasis by regulating ATP production and consumption 25. In 3T3-L1 cells, AMPK directly phosphorylates SREBP-1c at Ser372, inhibiting its proteolytic maturation and nuclear translocation 26. This suggests that the inhibition of lipid biosynthesis through the modulation of the AMPK-SREBP-1c signaling pathway may represent a promising therapeutic approach for obesity treatment.

In addition to these metabolic signaling pathways, oxidative stress is also considered a key factor influencing obesity. Oxidative stress has been implicated in obesity because excessive reactive oxygen species (ROS) can activate signaling pathways that promote adipogenesis and lipogenesis through regulators such as sterol regulatory element-binding protein-1c (SREBP-1c), CCAAT/enhancer-binding protein alpha (C/EBPα), and peroxisome proliferator-activated receptor gamma (PPARγ) 27, 28. Consequently, compounds with high antioxidant capacity have been shown to attenuate these processes, representing a complementary strategy for anti-obesity treatment 27, 29.

In this study, we evaluated the anti-obesity effects of ginger leaf subcritical water extract (GLE), highlighting its potential as a therapeutic agent for obesity. Multiple natural compounds have been reported to inhibit adipogenesis in a stage-dependent manner 30, 31. Based on these findings, we investigated the timing of GLE effects by analyzing expression levels of key markers at early (4 h and day 2) and terminal (day 7) stages of differentiation. Additionally, the molecular mechanisms underlying GLE effects were examined, with particular emphasis on the AMPK-SREBP-1c signaling pathway.

## Materials and Methods

### Preparation of GLE

Ginger leaves were obtained from a commercial supplier in Seosan, Chungcheongnam-do, Korea, and were provided as a homogeneous batch suitable for extraction. The leaves were dried in a 60 °C hot air dryer for 12 h, ground, and sieved through a 30-mesh to obtain a dry powder. Prior to extraction, the powder was spread on a petri dish and further dried at 60-70 °C for 24 h. A mixture of 1 g of ginger leaf powder and 2 g of diatomaceous earth was packed into a 22 mL stainless-steel extraction cell (23 mm i.d. × 50 mm, Dionex, Sunnyvale, CA, USA). Subcritical water extraction was carried out at 130 °C for 10 min under 10 MPa using a Dionex™ ASE™ 350 Accelerated Solvent Extractor (Dionex, Sunnyvale, CA, USA) with distilled water. The extract was centrifuged at 4,000 rpm for 25 min, and the supernatant was collected, frozen in an ultra-low-temperature freezer for 24 h, and lyophilized for 48 h using a freeze-dryer.

### Quantitative analysis of GLE bioactive compounds using high-performance liquid chromatography (HPLC)

HPLC analysis was performed using an Agilent 1100-diode array detector system. Separation was carried out on an Imtakt Cadenza CD-C18 column (4.6 × 250 mm, 3 μm) maintained at 35°C. A gradient system composed of formic acid in water (mobile phase A) and acetonitrile (mobile phase B) was delivered at a flow rate of 0.7 mL/min. The gradient elution program was as follows: 10% B (0-5 and 60-65 min), 80% B (5-55 min), 95% B (55-60 min). The injection volume was 10 μL, and the detection was performed at 350 nm.

### ABTS radical scavenging assay

A 7 mM solution of 2,2′-azino-bis(3-ethylbenzothiazoline-6-sulfonic acid) (ABTS; Sigma-Aldrich, MO, USA) was mixed with a 2.45 mM solution of potassium persulfate and incubated in the dark for 24 h. Subsequently, 150 µL of the ABTS solution was added to 50 µL of ascorbic acid (1.56-20 μg/mL) or GLE (12.5-1,000 μg/mL) at each concentration in a 96-well plate. The reaction was carried out in the dark for 5 min, and absorbance was measured at 734 nm using an Epoch microplate spectrophotometer (BioTek Instruments, Winooski, VT, USA). ABTS radical scavenging ability was calculated using the following formula:



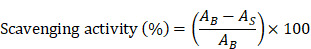



where A_B_ and A_S_ represent the absorbance values of the blank and sample, respectively.

### Cell culture and differentiation

3T3-L1 cells were obtained from the American Type Culture Collection (ATCC; Manassas, VA, USA). 3T3-L1 preadipocytes were cultured in Dulbecco's modified Eagle's medium (DMEM; Corning, NY, USA) supplemented with 10% bovine calf serum (Gibco, Grand Island, NY, USA) and 1% antibiotic-antimycotic solution (Gibco, Grand Island, NY, USA) in a 5% CO_2_ incubator at 37 °C.

To induce differentiation, 3T3-L1 preadipocytes were seeded at a density of 5 × 10^4^ cells/mL and maintained until reaching confluence. The cells were then cultured in DMEM containing 10% fetal bovine serum (FBS; Gibco, Grand Island, NY, USA), 0.5 mM 1-methyl-3-isobutylxanthine (Sigma-Aldrich, MO, USA), 1 μM dexamethasone (Sigma-Aldrich, MO, USA), and 10 μg/mL insulin (Gibco, Grand Island, NY, USA) for 3 days. This was followed by a 2-day incubation in DMEM containing 10% FBS and 10 μg/mL insulin. From day 5 onward, the medium was replaced with DMEM containing 10% FBS and maintained until day 7. GLE was administered during the differentiation period.

### Cell viability assay

Cell viability was evaluated using the 3-(4,5-dimethylthiazol-2-yl)-2,5-diphenyltetrazolium bromide (MTT; Sigma-Aldrich, MO, USA) assay. 3T3-L1 preadipocytes were seeded at a density of 1 × 10^5^ cells/mL in 96-well plates and incubated for 24 h, followed by treatment with GLE at six concentrations (0, 50, 100, 200, 400, and 800 µg/mL) for 72 h. Medium containing 0.5 mg/mL MTT solution was added to each well and incubated at 37°C for 1 h. After aspirating the medium, formazan crystals were solubilized in 100 μL of dimethyl sulfoxide, and absorbance was measured at 540 nm. 3T3-L1 adipocytes were differentiated for 7 days as previously described and subsequently subjected to the MTT assay using the same protocol.

### Oil red O staining

Lipid accumulation was assessed by Oil Red O staining. 3T3-L1 cells were seeded at a density of 5 × 10^4^ cells/mL in 6-well plates, differentiated for 7 days, and fixed with 4% paraformaldehyde (iNtRON Biotechnology, Gyeonggi-do, Korea). Oil Red O solution (Sigma-Aldrich, MO, USA), diluted in a 3:2 ratio with distilled water, was used to stain the cells. The stained lipid droplets of the cells were washed and imaged using an ECLIPSE Ts2 microscope (Nikon, Tokyo, Japan). After capturing the image, the staining dye from the cells was then eluted using 100% isopropanol (Sigma-Aldrich, MO, USA), and absorbance was measured at 500 nm using a microplate spectrophotometer.

### Quantitative reverse transcription polymerase chain reaction (qRT-PCR) analysis

Total mRNA was extracted from 3T3-L1 cells using the Total RNA Extraction Kit (iNtRON Biotechnology, Gyeonggi-do, Korea). mRNA quantified at 50 ng/µL was mixed with GoScript™ Reverse Transcriptase Mix (Promega, Madison, WI, USA) and reverse transcribed into cDNA using the TaKaRa PCR Thermal Cycler Dice® Touch (Takara Bio, Shiga, Japan). The synthesized cDNA was mixed with TB Green® Premix Ex Taq™ II (Takara Bio, Shiga, Japan) and forward and reverse primers (Table 1), followed by qRT-PCR using the QuantStudio 3 Real-Time PCR Instrument (Applied Biosystems, Foster City, CA, USA). Relative mRNA expression was determined using the 2⁻^ΔΔ^CT method.

### Western blot analysis

3T3-L1 cells were lysed in RIPA buffer (iNtRON Biotechnology, Gyeonggi-do, Korea) containing Halt™ phosphatase inhibitor cocktail (Thermo fisher scientific, Waltham, MA, USA) and a protease inhibitor (Sigma-Aldrich, MO, USA). After incubating for 10 min at 4 °C, the lysates were centrifuged at 13,000 rpm for 10 min. The supernatant was collected, and the protein was quantified using the TaKaRa BCA Protein Assay Kit (Takara Bio, Shiga, Japan). 40 μg of protein was electrophoresed on a sodium dodecyl sulfate-polyacrylamide gel and transferred to a polyvinylidene difluoride membrane. The membrane was blocked with 5% skim milk (BD Difco, Sparks, MD, USA) for 1 h and incubated with primary antibody (Table 2) at 4 °C overnight. The membranes were then incubated with horseradish peroxidase-conjugated secondary antibodies (Promega, Madison, WI, USA) for 1 h at room temperature. Protein bands were detected using Western Blot Detection System (iNtRON Biotechnology, Gyeonggi-do, Korea) and quantified with ImageQuant™ LAS 500 system (GE Healthcare Life Sciences, Little Chalfont, UK).

### Statistical analysis

All data are expressed as the mean ± standard deviation of at least three independent experiments. Statistical analysis was conducted using GraphPad Prism 10 software (GraphPad Software Inc., San Diego, CA, USA). Differences between two groups were evaluated using the Student's *t*-test, while comparisons among more than two groups were analyzed using one-way analysis of variance (ANOVA) followed by Tukey's post hoc test. A *p*-value < 0.05 was considered statistically significant.

## Results

### Phytochemical profile and ABTS radical scavenging activity of GLE

**Figure 1:**
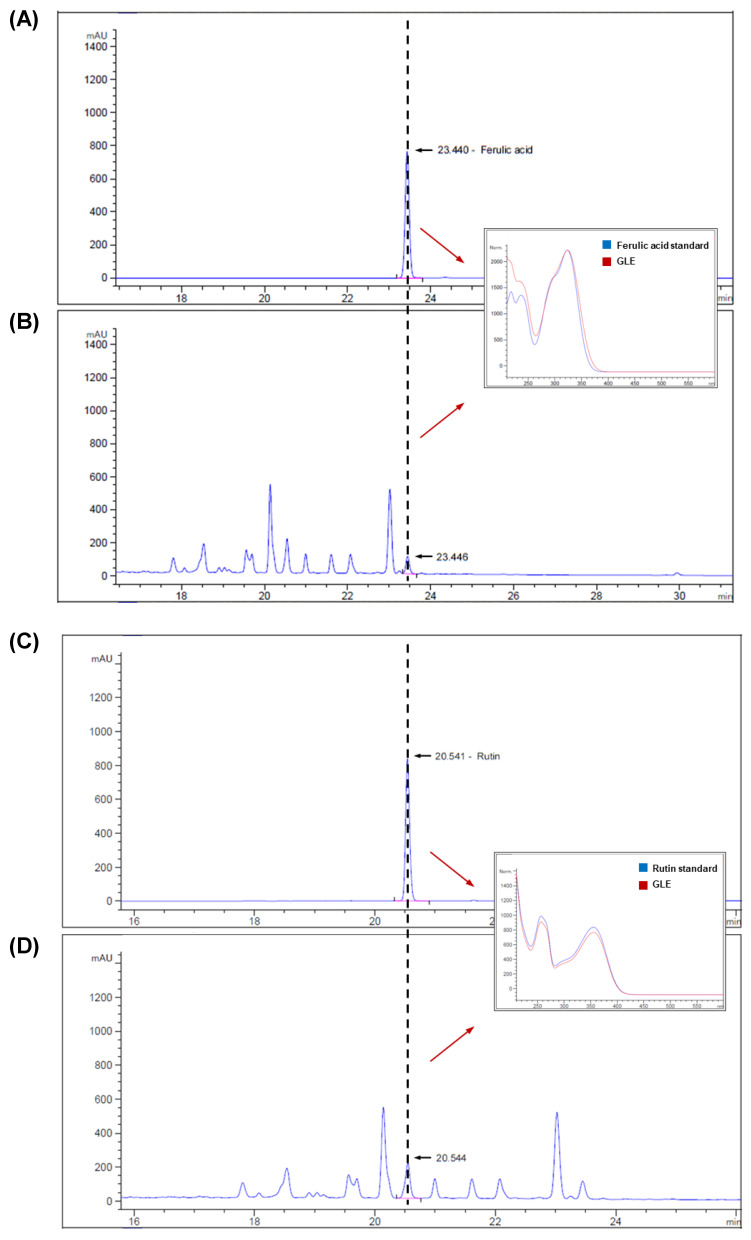
Quantification of bioactive compound in GLE using high-performance liquid chromatography (HPLC). (A) Ferulic acid standard (0.451 mg/mL) and (B) GLE sample. (C) Rutin standard (0.153 mg/mL) and (D) GLE sample. GLE, ginger leaf subcritical water extract

GLE contained two major phenolic compounds, ferulic acid (2.27 mg/g) and rutin (1.91 mg/g), as identified by HPLC analysis (Fig. 1). Given the documented antioxidant properties of these phenolics, the antioxidant capacity of GLE was further assessed using the ABTS radical-scavenging assay (Fig. S1). GLE exhibited a concentration-dependent scavenging effect, with approximately 90% radical scavenging at concentrations above 500 µg/mL. The IC_50_ value of GLE was 168.86 ± 8.98 µg/mL, whereas that of ascorbic acid, used as a positive control, was 8.40 ± 0.01 µg/mL.

### Effects of GLE on cell viability and lipid accumulation in 3T3-L1 cells

**Figure 2:**
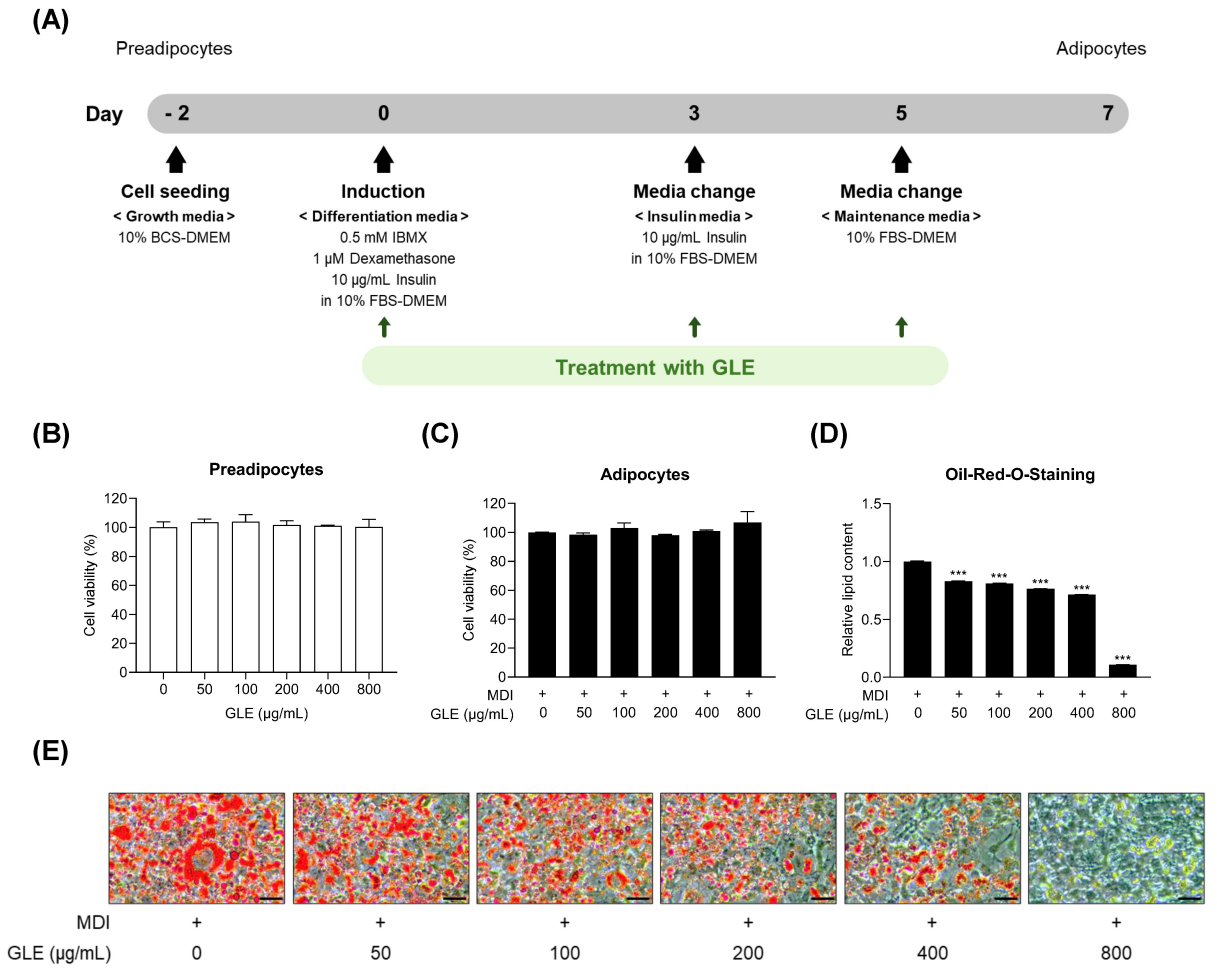
Effects of GLE on cell viability and lipid accumulation in 3T3-L1. (A) Experimental design for the differentiation of 3T3-L1 preadipocytes. Cell viability of (B) 3T3-L1 preadipocytes after 72 h and (C) 3T3-L1 adipocytes after 7 d. (D) Quantification of Oil Red O-stained lipid droplets. (E) Representative images of Oil Red O staining (200×; scale bar = 50 μm). Data are presented as the mean ± SD. ^***^p < 0.001 vs. 0 μg/mL. GLE, ginger leaf subcritical water extract

The protocol for the differentiation of 3T3-L1 preadipocytes into adipocytes is shown in Fig. 2A. 3T3-L1 preadipocytes and adipocytes were treated with GLE at concentrations of 50, 100, 200, 400, and 800 µg/mL to determine the non-cytotoxic concentration. GLE showed no cytotoxicity at concentrations up to 800 μg/mL in both preadipocytes and adipocytes (Fig. 2B, C). Differentiated 3T3-L1 cells were stained with Oil Red O to assess the effect of GLE treatment on lipid accumulation. Compared to untreated cells, all GLE-treated groups showed a significant reduction in lipid accumulation, with the most pronounced reduction observed at 800 μg/mL (Fig. 2D, E). These findings suggest that GLE reduces lipid production and accumulation in 3T3-L1 cells.

### Effects of GLE on adipogenesis in 3T3-L1 adipocytes

**Figure 3:**
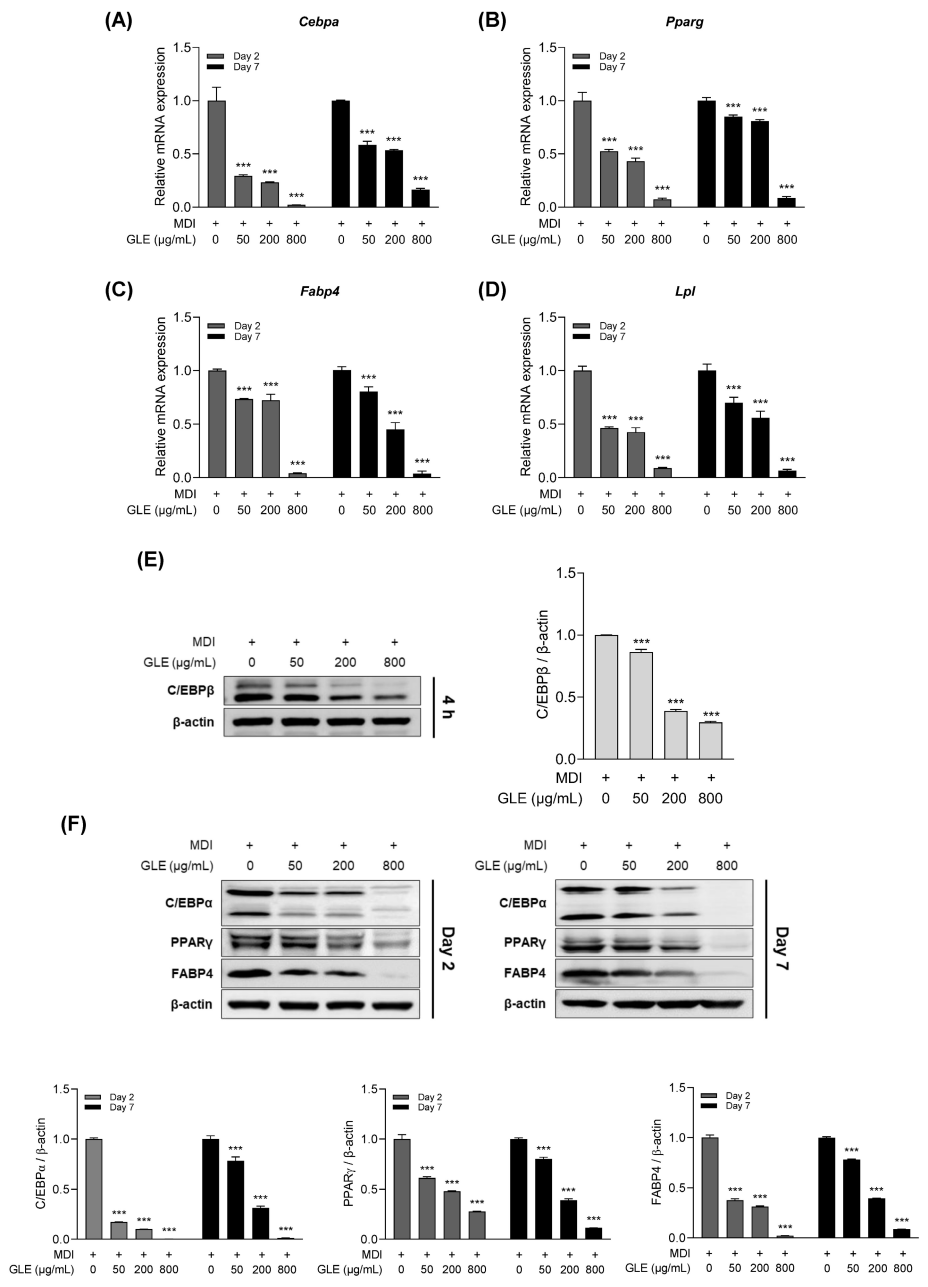
Effects of GLE on adipogenesis in 3T3-L1 adipocytes. Relative mRNA expression levels of (A) *Cebpa*, (B) *Pparg*, (C) *Fabp4*, and (D) *Lpl* on days 2 and 7. (E) Representative western blot images and quantification of C/EBPβ at 4 h. (F) Western blot images and quantification of C/EBPα, PPARγ, and FABP4 on days 2 and 7. Data are presented as the mean ± SD. ^***^p < 0.001 vs. 0 μg/mL. GLE, ginger leaf subcritical water extract

To assess whether GLE reduces adipogenesis in 3T3-L1 adipocytes, qRT-PCR and western blot analyses were performed. GLE treatment decreased the mRNA expression levels of C/EBPα, PPARγ, fatty acid binding protein 4 (FABP4), and lipoprotein lipase (LPL) on days 2 and 7 (Fig. 3A-D). In addition, GLE treatment reduced C/EBPβ protein expression at 4 h (Fig. 3E). Protein expression levels of C/EBPα, PPARγ, and FABP4 were also decreased by GLE treatment on days 2 and 7 (Fig. 3F). These results indicate that GLE inhibits C/EBPβ expression during the early stage of differentiation in 3T3-L1 cells, which in turn downregulates the expression of C/EBPα, PPARγ, FABP4, and LPL.

### Effects of GLE on lipogenesis in 3T3-L1 adipocytes

**Figure 4:**
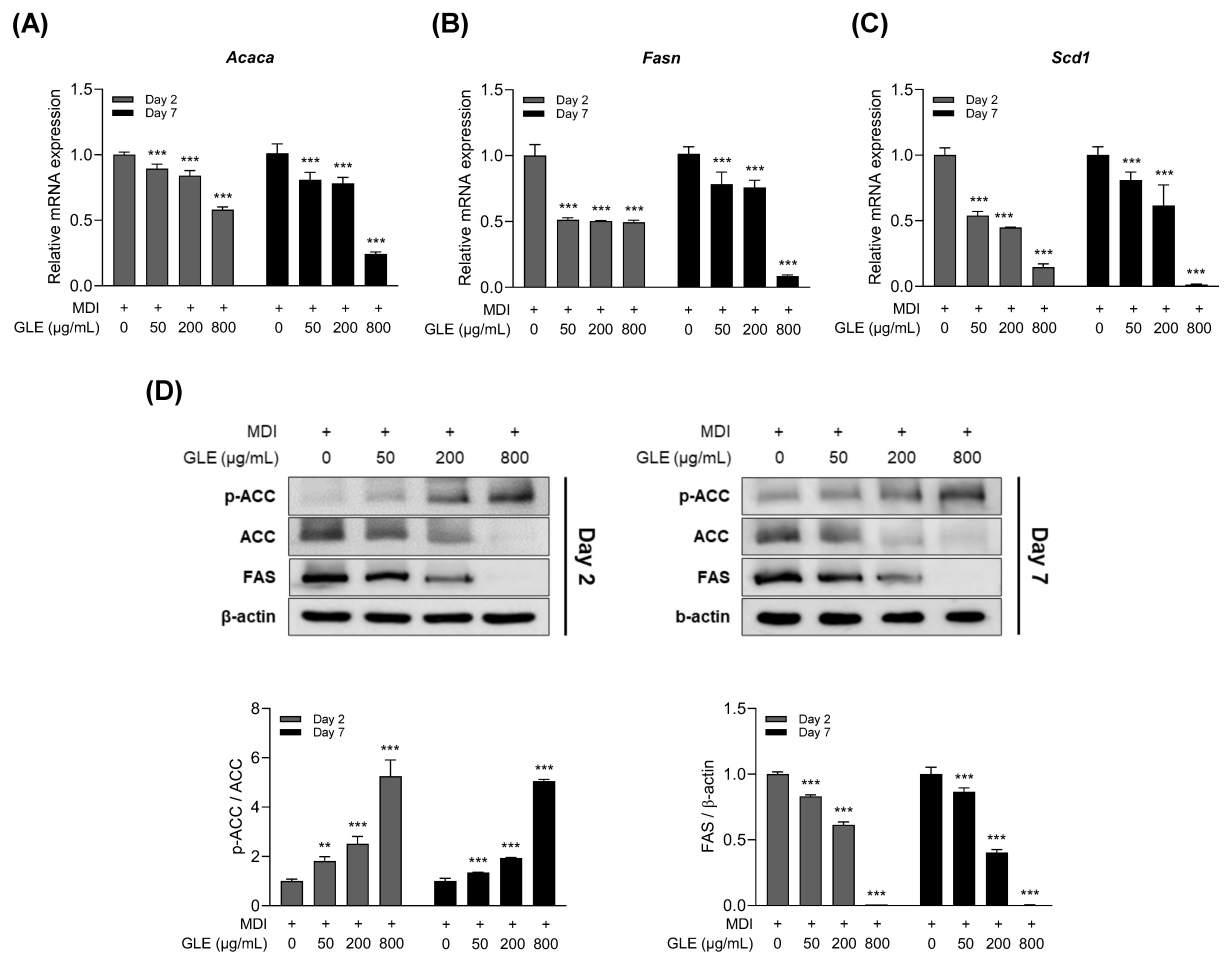
Effects of GLE on lipogenesis in 3T3-L1 adipocytes. Relative mRNA expression levels of (A) *Acaca*, (B) *Fasn*, and (C) *Scd1* on days 2 and 7. (D) Representative western blot images and quantification of ACC and FAS protein levels on days 2 and 7. Data are presented as the mean ± SD. ^**^p < 0.01, ^***^p < 0.001 vs. 0 μg/mL. GLE, ginger leaf subcritical water extract

To assess the effect of GLE on lipogenesis, the expression of lipogenesis-related factors was evaluated on days 2 and 7 of differentiation in 3T3-L1 adipocytes. GLE decreased the mRNA expression levels of acetyl-CoA carboxylase (ACC), fatty acid synthase (FAS), and stearoyl-CoA desaturase 1 (SCD1) (Fig. 4A-C). Western blot analysis revealed that GLE inhibited FAS protein expression, increased ACC phosphorylation, and decreased total ACC levels (Fig. 4D). These results indicate that GLE inhibited lipogenesis by modulating the expression of lipogenesis-related genes from day 2 of differentiation.

### Effects of GLE on the AMPK-SREBP-1c signaling pathway in 3T3-L1 adipocytes

**Figure 5:**
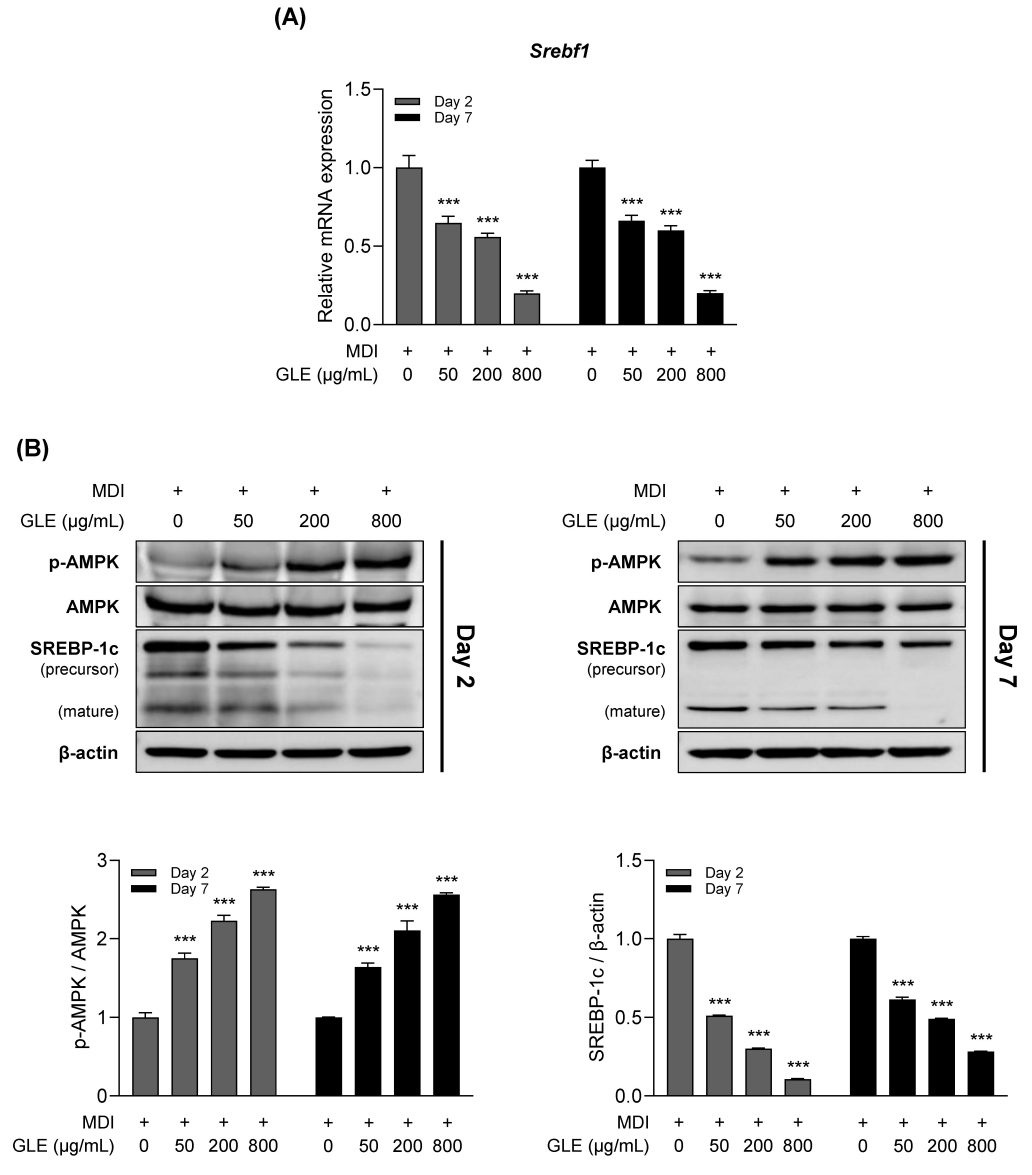
Effects of GLE on AMPK and SREBP-1c expression in 3T3-L1 adipocytes. (A) Relative mRNA expression of *Srebf1*. (B) Western blot images and quantification of p-AMPK/AMPK and SREBP-1c protein levels on days 2 and 7. Data are presented as the mean ± SD. ^***^p < 0.001 vs. 0 μg/mL. GLE, ginger leaf subcritical water extract

To determine whether AMPK and SREBP-1c, which are implicated in the regulation of adipogenesis and lipogenesis, mediate the anti-obesity effects of GLE, expression levels were evaluated on days 2 and 7 (Fig. 5A, B). GLE reduced the mRNA expression of SREBP-1c and decreased both the precursor and mature forms of the SREBP-1c protein. In addition, GLE upregulated AMPK phosphorylation.

**Figure 6:**
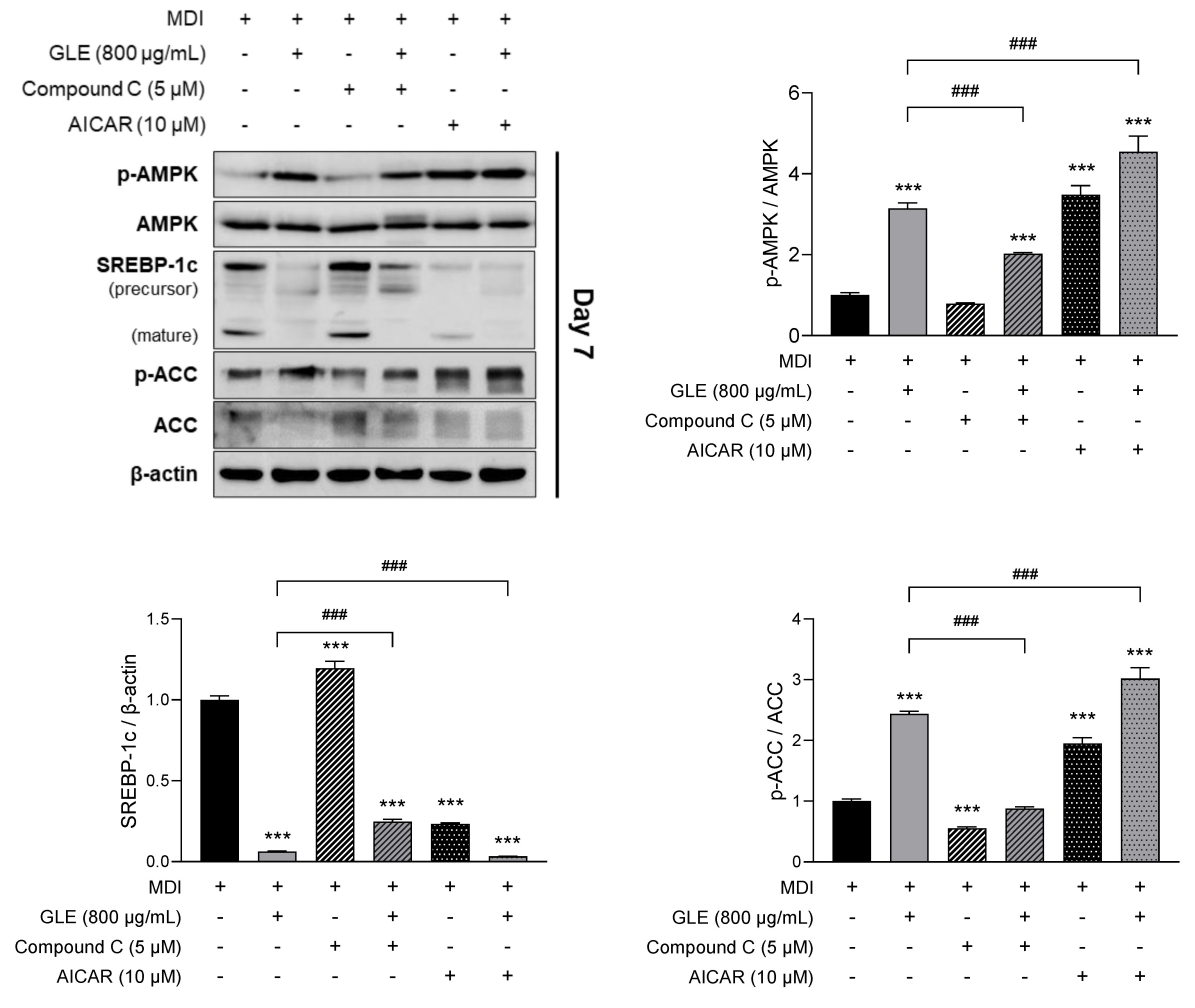
Effects of GLE on the AMPK**-**SREBP-1c signaling pathway in 3T3-L1 adipocytes. Western blot images and quantification of p-AMPK/AMPK, SREBP-1c, and p-ACC/ACC protein levels treated with or without Compound C (AMPK inhibitor), or AICAR (AMPK activator) on day 7. Data are presented as the mean ± SD. ^***^p < 0.001 vs. MDI; ^###^*p* < 0.001 vs. GLE. GLE, ginger leaf subcritical water extract

To further investigate whether GLE mediates its effects through the AMPK-SREBP-1c signaling pathway, 3T3-L1 cells were treated with GLE in the presence of AMPK modulators, including the AMPK inhibitor compound C and the AMPK activator AICAR. GLE increased phosphorylation of AMPK and ACC and decreased SREBP-1c expression. This effect was reversed by co-treatment with compound C (Fig. 6). Furthermore, the GLE-induced increase in AMPK and ACC phosphorylation and reduction in SREBP-1c expression were enhanced by co-treatment with AICAR. GLE also significantly reversed lipid accumulation and the expression of adipogenic markers such as C/EBPα, PPARγ, and FABP4 when co-treated with Compound C (Fig. S2). These findings indicate that GLE inhibits adipogenesis, lipogenesis, and lipid accumulation via the AMPK-SREBP-1c signaling pathway in 3T3-L1 adipocytes.

### Effects of GLE on phosphorylation of MAPKs in 3T3-L1 adipocytes

**Figure 7:**
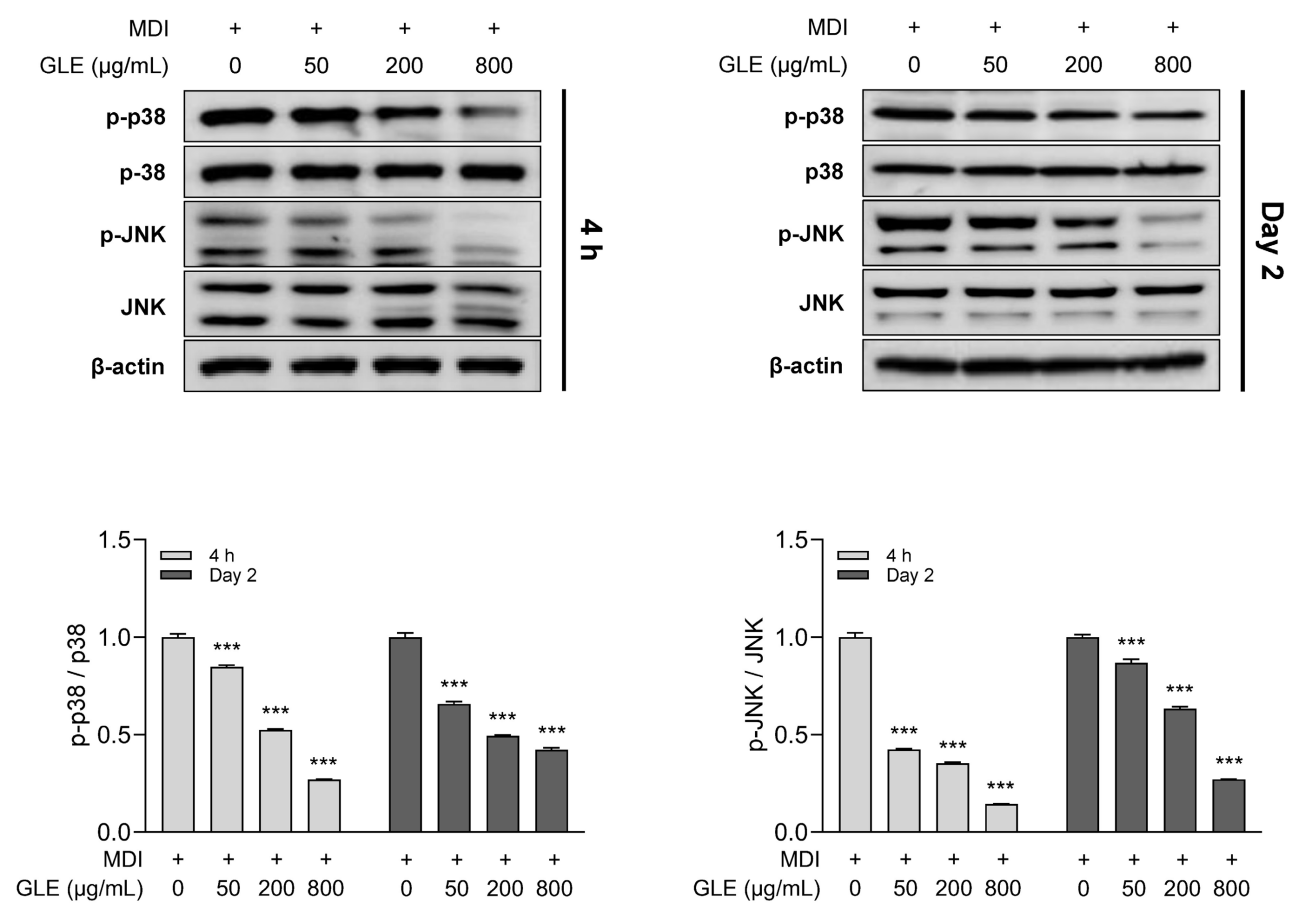
Effects of GLE on MAPK pathway inhibition in 3T3-L1 adipocytes. Western blot images and quantification of p-p38/p38 and p-JNK/JNK at 4 h and day 2. Data are presented as the mean ± SD. ^***^p < 0.001 vs. 0 μg/mL. GLE, ginger leaf subcritical water extract

Mitogen-activated protein kinases (MAPKs) regulate adipogenesis- and lipogenesis-related factors through multiple signaling pathways 32, 33. To evaluate the effect of GLE on MAPK activity, phosphorylation levels of key MAPKs were assessed. GLE treatment decreased the phosphorylation of p38 and c-Jun N-terminal kinase (JNK) in a concentration-dependent manner at both 4 h and day 2 (Fig. 7). These results suggest that GLE reduces p38 and JNK phosphorylation from the early stages of differentiation, indicating that GLE-mediated MAPKs inhibition may modulate the activity of key factors involved in adipogenesis and lipogenesis.

## Discussion

Obesity is a chronic disease characterized by an excessive accumulation of adipose tissue resulting from both hyperplasia and hypertrophy of adipocytes 34. It is not merely an individual condition but persists across the lifespan and transgenerationally 35. However, long-term drug treatment is associated with risks of dependence and adverse effects 36. Consequently, natural products have attracted interest as potentially safer therapeutic alternatives for managing obesity.

Among the parts of ginger, the rhizome has been extensively studied and is known to exhibit various bioactive properties 37, whereas the leaf is considered a by-product and has not been fully studied. Interestingly, previous studies comparing different parts of ginger have reported that the leaf exhibits the highest antioxidant capacity and polyphenol content among the rhizome, leaf, and flower 15, 38. This suggests the pharmacological potential of the ginger leaf. Consistent with these findings, GLE showed ABTS radical-scavenging activity in a concentration-dependent manner (Fig. S1) and was rich in polyphenols. HPLC analysis indicated high levels of ferulic acid and rutin. Ferulic acid is a plant-derived phenolic compound commonly found in leaf and legumes 39, and rutin is a plant-derived flavonoid that is both non-toxic and non-oxidizing, which makes it more favorable to use than other flavonoids 40. Polyphenolic compounds have been reported to protect cells against oxidative stress due to their antioxidant properties, thereby exerting anti-obesity effects. Both ferulic acid and rutin exhibit multiple physiologically active properties, including antioxidant and anti-obesity effects 41, 42. Previous studies have demonstrated that administration of ferulic acid to high-fat diet-induced obese mice reduced body fat accumulation and body weight, thereby decreasing the risk of obesity and metabolic syndrome 43, 44. Rutin has been shown to ameliorate obesity by suppressing adipocyte differentiation and the expression of adipogenesis-related transcription factors, as well as regulating body weight in 3T3-L1 cells and obesity-induced mice. This supports the idea that the bioactive compounds in GLE may exert anti-obesity effects, in part, through their antioxidant properties 45, 46. The ABTS radical-scavenging result reflects chemical antioxidant capacity and was not interpreted as evidence of intracellular redox modulation.

Subcritical water refers to liquid water maintained below its critical temperature (374.15°C) and pressure (22.1 MPa). In this state, it exhibits distinct physicochemical properties that facilitate the dissolution of both polar and nonpolar compounds. Its low viscosity and surface tension, along with a high diffusion coefficient, enhance mass transfer efficiency 47. Additionally, subcritical water remains relatively stable at elevated temperatures, which helps preserve thermolabile bioactive constituents 48. Unlike conventional extraction techniques, subcritical water extraction is solvent-free, achieving high extraction efficiency while mitigating solvent-related risks. Despite these advantages, no studies have systematically examined the composition or bioactivities of ginger leaf extracts obtained via subcritical water extraction. In screening, we compared extracts from subcritical water, 70% ethanol, water, and dry powder. Both the water and 70% ethanol extracts showed high cytotoxicity. The dry powder was safe but showed no anti-adipogenic effect. The subcritical water extract was the only one that satisfied both essential criteria: high safety and potent lipid inhibition. Therefore, we selected subcritical water extract for this study (data not shown). In this study, we investigated the anti-obesity potential of GLE by evaluating its effects on adipogenesis and lipogenesis in 3T3-L1 cells and elucidated the mechanisms involved in these processes.

First, we investigated whether GLE affects the differentiation of preadipocytes into adipocytes. C/EBPα functions as a master regulator of adipose tissue development and promotes adipocyte differentiation 38, 49. Additionally, PPARγ, a transcription factor of the nuclear receptor superfamily, is a key regulator of fat and glucose metabolism and storage 50, 51. These two critical factors interact during adipogenesis to activate adipogenesis-specific genes, such as FABP4 and LPL 21, 52. GLE inhibited the expression of C/EBPα, PPARγ, and their downstream targets, FABP4 and LPL, on days 2 and 7 of differentiation. FABP4 is a well-established late adipogenic marker whose expression markedly increases during the later stages of differentiation; therefore, its strong expression on day 7 and the reduction observed with GLE reflect inhibition of late-stage adipocyte maturation. In addition, we hypothesized that GLE reduced the expression of adipogenesis-related genes by initially inhibiting C/EBPβ. C/EBPβ plays a crucial role in MCE and is responsible for the transactivation of the C/EBPα and PPARγ genes 53. C/EBPβ is expressed immediately after the initiation of adipogenesis, reaching peak levels within 4 h 54. GLE reduced C/EBPβ expression in 3T3-L1 cells at 4 h, suggesting that GLE inhibits adipogenesis at an early stage.

The MAPK pathway regulates adipogenesis throughout the entire differentiation process 32. In particular, p38 MAPK promotes adipocyte differentiation by directly activating C/EBPβ during the early stages of differentiation 55. However, p38 MAPK activity declines after 4 days of differentiation, indicating that its function is primarily restricted to early adipocyte differentiation stages 56. This temporal specificity suggests that targeting p38 MAPK signaling during early differentiation may be an effective strategy to regulate adipogenesis. Similar to p38, JNK has also been attributed an essential regulatory role limited to the early stages of adipocyte differentiation 57. We found that GLE inhibited phosphorylation of both p38 and JNK at 4 h and day 2 of differentiation, suggesting that GLE regulates adipogenesis from the outset by targeting the p38-C/EBPβ pathway and JNK. Nevertheless, the role of JNK in adipogenesis remains controversial, warranting further investigation to clarify the mechanisms of JNK activity in adipocytes.

Lipogenesis includes the synthesis of FAs and TGs 58. FA synthesis is governed by the sequential transcription of ACC, FAS, and SCD1, which are key regulators of lipogenesis 59. ACC catalyzes the carboxylation of acetyl-CoA to malonyl-CoA, the substrate for FAS. SCD1 catalyzes the conversion of saturated fatty acids (SFAs) into monounsaturated fatty acids (MUFAs), which are essential for the synthesis of TGs, cholesterol esters, and very low-density lipoproteins (VLDL) 60, 61. In particular, MUFAs are preferentially utilized for TG synthesis compared with SFAs 62, 63. Therefore, SCD1 plays a key role in lipid metabolism and has been proposed as a potential therapeutic target for obesity. GLE reduced the total ACC expression level and increased ACC phosphorylation. The increase in ACC phosphorylation serves as a short-term off-switch to immediately inactivate the existing ACC enzyme. This is the direct result of AMPK activation 64. Simultaneously, the decrease in total ACC expression is a long-term machinery reduction. By inhibiting the main adipogenic pathway, such as SREBP-1c 65, GLE reduces the synthesis of new ACC protein. Therefore, GLE provides robust, dual-regulation: it shuts off the enzyme you have via phosphorylation and reduces the expression of the enzyme. GLE also decreased the expression levels of FAS and SCD1. These results suggest that GLE inhibits FA synthesis in 3T3-L1 cells by modulating these key enzymes. Beyond its role in FA synthesis, SCD1 broadly regulates lipid metabolism 63. A previous study demonstrated that inhibition of SCD1 in adipocytes was linked to activation of AMPK and modulation of SREBP-1c and PPARγ expression 66. However, the mechanism by which SCD1 influences GLE-induced AMPK activation in 3T3-L1 cells requires further investigation.

The lipid metabolism effects of SREBP-1c have been extensively investigated. SREBP-1c promotes adipogenesis by activating PPARγ ligands 67. It regulates transcriptional activity by directly binding to the promoters of lipogenic enzymes such as ACC and FAS, and its inhibition improves insulin resistance and reduces lipid accumulation via the FAS/ACC pathway 65, 68. These findings collectively indicate that SREBP-1c functions as a transcription factor involved in both adipogenesis and lipogenesis. SREBP-1c is synthesized as an inactive precursor in the endoplasmic reticulum (ER), where it forms a complex with SREBP cleavage-activating protein 69. Its activation requires two sequential proteolytic cleavage steps 70. The amino-terminal domain of SREBP-1c subsequently translocates to the nucleus, where it induces the expression of genes involved in lipid metabolism. This activation process of SREBP-1c is directly regulated by AMPK. Phosphorylated AMPK binds to the Ser372 residue of SREBP-1c in the ER and phosphorylates SREBP-1c 26, 71. Phosphorylated SREBP-1c fails to undergo proteolytic cleavage and nuclear translocation, and therefore remains membrane-bound 26. AMPK phosphorylation is thus considered a master regulatory mechanism in lipid metabolism 72. In our study, GLE modulated adipogenesis and lipogenesis via the AMPK-SREBP-1c pathway in 3T3-L1 adipocytes. Compound C reversed the effects of GLE on AMPK phosphorylation and mature SREBP-1c expression, indicating that GLE suppressed SREBP-1c activation through AMPK signaling. GLE also reduced precursor SREBP-1c expression, consistent with previous findings that phosphorylated AMPK leads to decreased precursor SREBP-1c levels 71, 73. These results demonstrate that the AMPK-SREBP-1c pathway plays a key role in the anti-obesity effects of GLE in 3T3-L1 cells. Notably, combined treatment with AICAR, an AMPK activator, and GLE resulted in enhanced anti-obesity effects, suggesting that cooperative activation of AMPK may produce more effective outcomes for obesity treatment. While Compound C robustly suppressed AMPK- SREBP-1c signaling, its effects on adipogenic transcription factors and terminal differentiation markers were partial during the late differentiation stage. This dissociation suggests that AMPK plays a contributory, but not exclusive, role in regulating adipogenesis.

Finally, in our study, GLE significantly inhibited adipogenesis and lipogenesis in a dose-dependent manner (50-800 μg/mL). Notably, the effective starting concentration of GLE (50 μg/mL) was comparable to that reported for ginger rhizome extracts (25-50 μg/mL) in previous studies 74, 75. Lower concentrations of GLE (up to 200 μg/mL) significantly modulated adipogenic and lipogenic markers, while a strong reduction in lipid accumulation was observed at 800 μg/mL. This suggests that although lower concentrations are sufficient for molecular modulation, a higher concentration is necessary to produce a significant phenotypic effect.

## Conclusion

**Figure 8:**
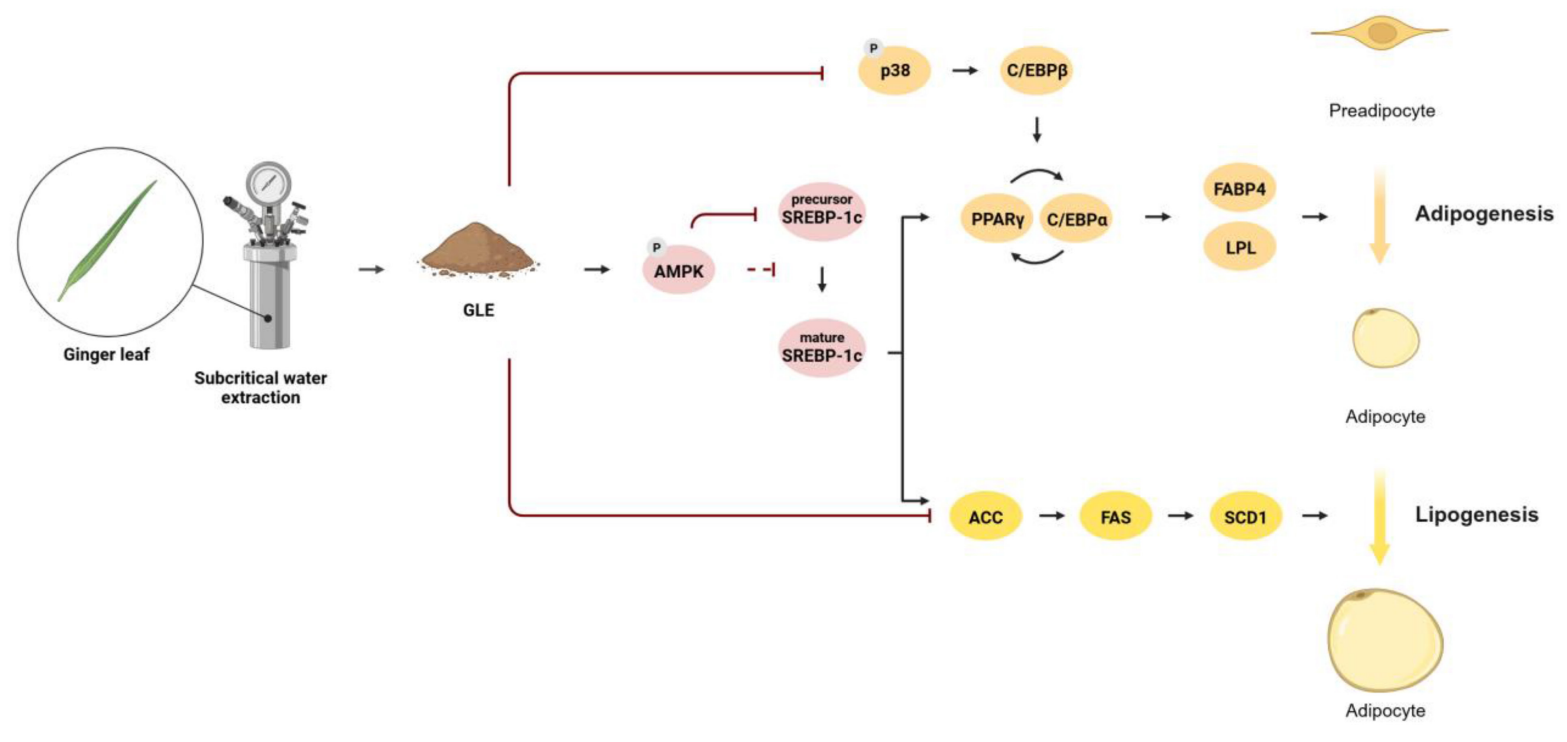
GLE inhibits adipogenesis, lipogenesis, and lipid accumulation through the AMPK-SREBP-1c signaling pathway and regulation of MAPKs

This study demonstrated that GLE inhibits adipogenesis, lipogenesis, and lipid accumulation through the AMPK-SREBP-1c signaling pathway and regulation of MAPKs (Fig. 8). Mechanistically, GLE suppressed the early transcription of adipogenesis- and lipogenesis-related genes at 4 h and day 2 following hormonal induction, with sustained downregulation observed through day 7 of differentiation. These findings highlight the potential of ginger leaf, a byproduct, as a functional ingredient for regulating lipid metabolism. However, further in vivo and clinical studies are required to validate the anti-obesity effects of GLE.

## Supplementary Material

Supplementary figures.

## Figures and Tables

**Table 1:** Primer sequences used for qRT-PCR analysis

Gene	Primer sequences (5′-3′)	Accession Number
*Srebf1*	F: TGTTGGCATCCTGCTATCTG R: AGGGAAAGCTTTGGGGTCTA	BC056922.1
*Acaca*	F: GCGTCGGGTAGATCCAGTT R: CTCAGTGGGGCTTAGCTCTG	NM_133360.3
*Fasn*	F: TTGCTGGCACTACAGAATGC R: AACAGCCTCAGAGCGACAAT	NM_007988.3
*Scd1*	F: TTCCCTCCTGCAAGCTCTAC R: CAGAGCGCTGGTCATGTAGT	NM_009127.4
*Pparg*	F: TTTTCAAGGGTGCCAGTTT R: AATCCTTGGCCCTCTGAGAT	U01841.1
*Cebpa*	F: TTACAACAGGCCAGGTTTCC R: GGCTGGCGACATACAGTACA	NM_007678.4
*Fabp4*	F: TCACCTGGAAGACAGCTCCT R: AATCCCCATTTACGCTGATG	NM_001409514.1
*Lpl*	F: TCCAAGGAAGCCTTTGAGAA R: CCATCCTCAGTCCCAGAAAA	NM_008509.2
*Cebpb*	F: CCAAGAAGACGGTGGACAA R: CAAGTTCCGCAGGGTGCT	NM_001287739.1
*Actb*	F: CCACAGCTGAGAGGAAATC R: AAGGAAGGCTGGAAAAGAGC	NM_007393.5

*Srebf1*, sterol regulatory element-binding protein 1; *Acaca*, acetyl-CoA carboxylase alpha; *Fasn*, fatty acid synthase; *Scd1*, stearoyl-CoA desaturase 1; *Pparg*, peroxisome proliferator-activated receptor gamma; *Cebpa*, CCAAT/enhancer-binding protein alpha; *Fabp4*, fatty acid-binding protein 4; *Lpl*, lipoprotein lipase; *Cebpb*, CCAAT/enhancer-binding protein beta; *Actb*, beta-actin

**Table 2:** Antibodies used for western blot analysis

Antibody	Source	Manufacturer	Catalog Number
C/EBPβ	Rabbit	Cell Signaling Technology	3087S
C/EBPα	Rabbit	Cell Signaling Technology	2295S
PPARγ	Rabbit	Cell Signaling Technology	2435S
FABP4	Rabbit	Cell Signaling Technology	2120S
Phospho-ACC	Rabbit	Cell Signaling Technology	3661S
ACC	Rabbit	Cell Signaling Technology	3662S
FAS	Mouse	Santa Cruz	SC-48357
Phospho-AMPK	Rabbit	Cell Signaling Technology	2535S
AMPK	Rabbit	Cell Signaling Technology	2532S
SREBP-1c	Mouse	Santa Cruz	SC-13551
Phospho-p38	Rabbit	Cell Signaling Technology	4511S
p38	Rabbit	Cell Signaling Technology	8690S
Phospho-JNK	Rabbit	Cell Signaling Technology	4668S
JNK	Rabbit	Cell Signaling Technology	9252S
β-actin	Mouse	Cell Signaling Technology	3700S

C/EBP, CCAAT/enhancer-binding protein; PPARγ, peroxisome proliferator-activated receptor gamma; FABP4, fatty acid-binding protein 4; ACC, acetyl-CoA carboxylase; FAS, fatty acid synthase; AMPK, adenosine monophosphate-activated protein kinase; FAS, fatty acid synthase; SREBP-1c, sterol regulatory element-binding protein 1c; JNK, c-Jun N-terminal kinase; β-actin, beta-actin

## Abbreviations

ABTS: 2,2′-azino-bis(3-ethylbenzothiazoline-6-sulfonic acid)
ACC: Acetyl-CoA carboxylase
AMPK: AMP-activated Protein Kinase
ANOVA: Analysis of Variance
C/EBP: CCAAT/enhancer-binding protein
DMEM: Dulbecco's Modified Eagle's Medium
FABP4: Fatty acid binding protein 4
FAS: Fatty acid synthase
FAs: Fatty acids
FBS: Fetal bovine serum
GLE: Ginger leaf subcritical water extract
HPLC: High-performance liquid chromatography
JNK: c-Jun N-terminal kinase
LPL: Lipoprotein lipase
MAPKs: Mitogen-activated protein kinases
MCE: Mitotic clonal expansion
MTT: 3-(4,5-dimethylthiazol-2-yl)-2,5-diphenyltetrazolium bromide
PPARγ: Peroxisome proliferator-activated receptor gamma
SCD1: Stearoyl-CoA desaturase 1
SREBP-1c: Sterol regulatory element-binding protein 1c
TGs: Triglyceride
