# Supplementary material for: Zingiber officinale Leaf Subcritical Water Extract Suppresses Adipogenesis and Lipogenesis in 3T3-L1 Adipocytes via the AMPK-SREBP-1c Signaling Pathway
Source: Int J Med Sci. 2026 Jun 4;23(7):2408–22. doi: 10.7150/ijms.121198 (PMC13280736; doi:10.7150/ijms.121198)
Supplement: Supplementary file 1 — Supplementary figures. [file ijmsv23p2408s1.pdf]

Supplementary Figures

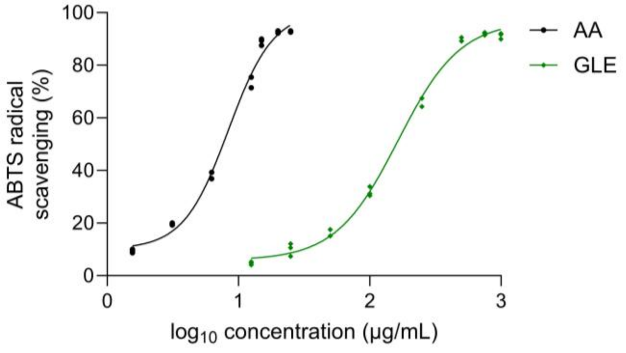

Fig S1. Antioxidant activity of GLE measured using the ABTS assay. GLE, ginger leaf subcritical water extract; AA, ascorbic acid

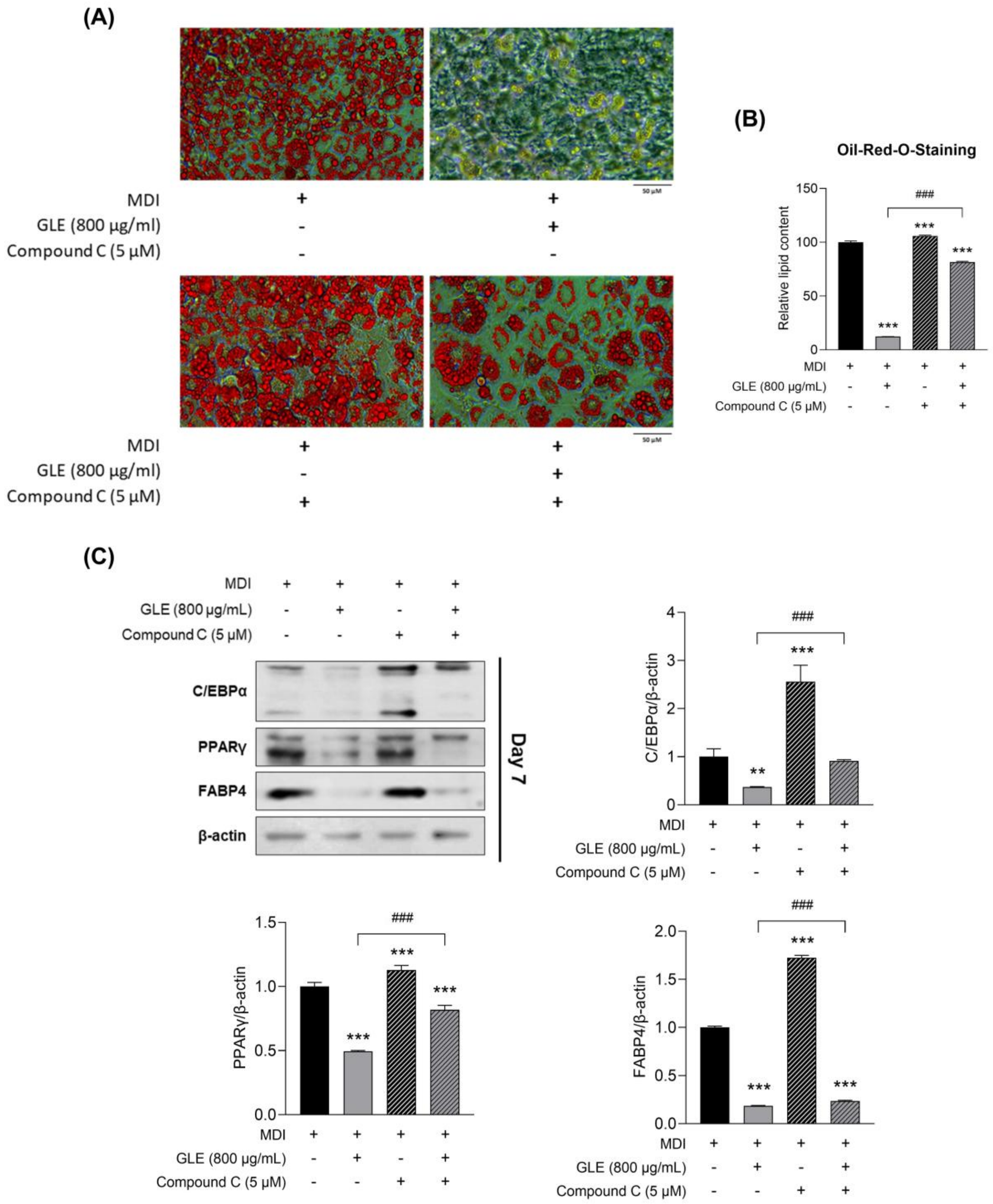

Fig S2. Effect of GLE on downstream target expression via the AMPK pathway. (A) Representative images of Oil Red O staining (200×; scale bar = 50 μm). (B) Quantification of Oil Red O-stained lipid droplets. (C) Western blot images and quantification of C/EBPα, PPARγ, and FABP4 protein levels treated with or without Compound C (AMPK inhibitor) on day 7. Data are presented as the mean ± SD. $^{**}p < 0.01$, $^{***}p < 0.001$ vs. MDI; $^{\#\#\#}p < 0.001$ vs. GLE. GLE, ginger leaf subcritical water extract
